# RT-RPA-PfAgo System: A Rapid, Sensitive, and Specific Multiplex Detection Method for Rice-Infecting Viruses

**DOI:** 10.3390/bios13100941

**Published:** 2023-10-20

**Authors:** Yan Liu, Wenqiang Xia, Wei Zhao, Peiying Hao, Zhengliang Wang, Xiaoping Yu, Xuping Shentu, Kai Sun

**Affiliations:** 1 Zhejiang Provincial Key Laboratory of Biometrology and Inspection & Quarantine, College of Life Sciences, China Jiliang University, Hangzhou 310018, China; liuyann@cjlu.edu.cn (Y.L.); s22020804084@cjlu.edu.cn (W.Z.); haopy@cjlu.edu.cn (P.H.); wzl@cjlu.edu.cn (Z.W.); yxp@cjlu.edu.cn (X.Y.); stxp@cjlu.edu.cn (X.S.); 2 Institute of Crop Science, College of Agriculture and Biotechnology, Zhejiang University, Hangzhou 310058, China; wqxia.zj@zju.edu.cn

**Keywords:** virus detection, plant pathogens, RT-RPA, PfAgo, rice virus

## Abstract

The advancement in CRISPR-Cas biosensors has transmuted the detection of plant viruses owing to their rapid and higher sensitivity. However, false positives and restricted multiplexing capabilities are still the challenges faced by this technology, demanding the exploration of novel methodologies. In this study, a novel detection system was developed by integrating reverse transcriptome (RT) techniques with recombinase polymerase isothermal amplification (RPA) and *Pyrococcus furiosus* Argonaute (PfAgo). The RT-RPA-PfAgo system enabled the simultaneous detection of rice ragged stunt virus (RRSV), rice grassy stunt virus (RGSV), and rice black streaked dwarf virus (RBSDV). Identifying targets via guide DNA without being hindered by protospacer adjacent motif sequences is the inherent merit of PfAgo, with the additional advantage of it being simple, cost-effective, and exceptionally sensitive, with detection limits between 3.13 and 5.13 copies/µL, in addition to it effectively differentiating between the three distinct viruses. The field evaluations were also in accordance with RT-PCR methods. The RT-RPA-PfAgo system proved to be a robust, versatile, highly specific, and sensitive method with great potential for practicality in future plant virus diagnostics.

## 1. Introduction

Rice (*Oryza sativa*) has been regarded as one of the world’s crucial crops, serving as a primary nutritional source for half of the global population. The regions providing a major share in rice cultivation, including China, Japan, Korea, India, Philippines, Vietnam, and other Southeast Asian countries, face challenges of different viruses affecting crop yields [1]. The major viral strains include rice stripe virus (RSV), rice grassy stunt virus (RGSV), rice dwarf virus (RDV), rice ragged stunt virus (RRSV), rice black streaked dwarf virus (RBSDV), southern rice black-streaked dwarf virus (SRBSDV), rice gall dwarf virus (RGDV), and rice stripe mosaic virus (RSMV) [2,3], transmitted predominantly by planthoppers and leafhoppers. Pest proliferation in the field can foster complex viral infections, precipitating intricate disease manifestations resulting in substantial yield losses.

Rice virus management poses a significant challenge due to the unavailability of specific antivirals, which demand early field detection. Currently, predominant approaches for detecting rice viruses encompass electron microscopy [4], serological assays [5], and molecular biology techniques like reverse transcription polymerase chain reaction (RT-PCR) [6] and real-time qPCR [7]. Serological methods, while widely used, often suffer from lower sensitivity, leading to frequent false negatives. Both electron microscopy and advanced molecular techniques necessitate the use of specialized, costly equipment such as electron microscopes and thermal cyclers, along with skilled laboratory personnel, constraining their widespread application. The advent of nucleic acid-based diagnostics has introduced innovative methods like recombinase polymerase amplification (RPA) and loop-mediated isothermal amplification (LAMP) assays. These assays, typically conducted with specifically designed primers recognizing unique sequences of the target, operate under isothermal conditions, obviating the need for thermal cycling. LAMP, in particular, has proven effective in identifying various rice viruses, including RRSV, RBSDV, and RGSV [8,9,10]. Although both RPA and LAMP are apt for field assays, their heightened sensitivity makes them prone to contamination, and they face difficulties in executing single-tube multiplex detection in field settings.

The clustered, the regularly interspaced short palindromic repeats (CRISPR)-associated Cas system has lately gained scientific attention as a potent technique for nucleic acid detection diagnostics, proficiently overcoming the limitations associated with isothermal amplification techniques. The platforms developed based on CRISPR-Cas13a’s SHERLOCK [11] and CRISPR-Cas12a’s DETECTOR [12] have been shown to exhibit rapid, sensitive, and specific detection of a broad spectrum of pathogens, including viral RNA and DNA, in point-of-care environments; thus, these have received immense attention in the field of viral molecular diagnostics. Nevertheless, CRISPR-Cas detection technologies have been associated with various constraints, like restricted detection sequences by protospacer adjacent motifs (PAM) or protospacer flanking sequences (PFS) and problems related to multiplex target detection [13].

Argonaute (Ago) proteins, widely distributed across many biological systems, have been reported to possess potential in nucleic acid detection [14]. Based on their biological origin, Ago proteins, as nucleic-acid-guided endonucleases, are categorized into eukaryotic Argonaute (eAgo) and prokaryotic Argonaute (pAgo). eAgo has been extensively studied for being involved in the RNA interference (RNAi) pathway [15,16], while pAgo has attracted immense attention from scientists due to its varied classifications and behaviors across different hosts. Moreover, certain pAgo proteins have shown several benefits compared to Cas nucleases owing to their capability of sequence-specific binding and cleavage of target DNA [17] and RNA [18,19]. They are not constrained by PAM sequence presence within the target DNA, thus providing increased flexibility in target nucleic acid selection [20]. In contrast to Cas nucleases requiring long RNA guides, most pAgo proteins employ short DNA molecules as a guide. Given the economic and stability advantages of DNA synthesis over RNA, it promotes the development of Ago-based nucleic acid detection systems. Due to their lower molecular size, the modification and production of these are relatively easy compared to Cas9. Moreover, they also offer cleaving of specific sequences of substrates, enabling the detection of multiple targets, which have been translated into various novel nucleic acid detections; *Thermus thermophilus* Argonaute (TtAgo), for example, has been engineered to enrich rare nucleic acids following PCR amplification [21]. Similarly, in the field of viral detection, *Pyrococcus furiosus* Argonaute (PfAgo) systematic cleavage has been exploited to facilitate sequence-specific detection of various viruses, like human papillomavirus, SARS-CoV-2, and influenza viruses [22,23,24].

Previous reports have demonstrated the pAgo potential for rapid and on-site detection of multiple human viruses; their applications in detecting plant RNA viruses have not been reported. Therefore, this study aimed to develop a highly specific and sensitive method by combining PfAgo and isothermal RPA amplification to concurrently detect multiple viruses, including RRSV, RGSV, and RBSDV, which is envisaged to outperform RT-PCR regarding specificity, sensitivity, and reproducibility. This innovative technique holds promise for its potential applicability across various plant viruses, enabling expedited identification of viral nucleic acid sequences.

## 2. Materials and Methods

### 2.1. Sample Collection and RNA Extraction

Rice plant samples displaying symptoms including chlorosis, stunting, and leaf malformation were collected from Hunan, Shanghai, and Zhejiang provinces in P.R. China during 2020–2022 and were stored at −80 °C until further use. The samples were subjected to total RNA extraction using a TaKaRa MiniBEST Plant RNA Extraction Kit (Takara, Dalian, China), followed by RNA quantification using a NanoDrop Lite Spectrophotometer (Thermo Fisher Scientific, Waltham, MA, USA).

### 2.2. Expression and Purification of PfAgo

A recombinant plasmid designed for PfAgo expression was derived from a previously studied protein sequence [24], enabling the expression of the N-terminal His-tagged PfAgo protein in *Escherichia coli* cells. Following expression, the PfAgo proteins were purified using Ni-affinity chromatography, a conventional method for His-tagged proteins. The final PfAgo protein exhibited an approximate purity of 90%, as confirmed by sodium dodecyl sulfate-polyacrylamide gel electrophoresis (SDS-PAGE) Figure S1.

### 2.3. Primers, gDNA, and Probes Design

The RPA primers, gDNA, and probes were synthesized by Genscript Biotechnology (Nanjing, China) and are shown in Table S1. The RPA primers were designed to match the specific sequences of the different viruses following the manufacture’s specific parameters (TwistDx, Cambridge, UK). Specifically, the RRSV-RPA primers (F/R) were tailored to amplify the S8gp1 gene fragment, spanning nucleotides 684–987 of the RRSV segment 8 (GenBank Accession NC_003758.1). The RBSDV-RPA primers (F/R) targeted the ORF1 gene fragment, covering nucleotides 925–1250, from the RBSDV segment 10 (GenBank Accession NC_003733.1). Meanwhile, the RGSV-RPA primers (F/R) aimed to amplify the Pc5 gene fragment, ranging from nucleotides 1614 to 1892, found in the RGSV segment 5 (GenBank Accession NC_003733.1). The gDNA design was based on the PfAgo cleaving ability of DNA complementary to its bases, mediated by ssDNA with a phosphate group at the 5′ end. Moreover, the probe was designed as an ssDNA sequence complementary to the newly generated gDNA, flanked by a fluorophore (e.g., carboxyfluorescein (FAM), Victoria (VIC), or rhodamine-X (ROX) and a quencher (e.g., black hole quencher-1 or 2 (BHQ1 or -2)).

### 2.4. Preparation of RNA Standards

The RNA standards for sensitivity analysis were synthesized via in vitro transcription. Briefly, the virus-specific sequences were amplified through RT-PCR initially using primers listed in Table S1, followed by validating amplified target sequences employing agarose gel electrophoresis, subsequently purified, and integrated into PMD-18T vectors (Takara, Dalian, China) through TA cloning. The standard RNA was then synthesized using the T7 in vitro transcription systems (Riboprobe System T7; Promega, Madison, WI, USA), adopting the previously reported method [25]. The detection threshold was assessed in triplicate, employing a 10-fold serial dilution of the transcribed RNA standards (ranging from 10^8^ to 10^0^ copies/reaction) as templates for the RT-PRA-PfAgo assays.

### 2.5. RT-PRA Reaction

The RT-RPA reaction was conducted using the TwistAmp Basic RT RPA Kit (TwistDx, Cambridge, UK) per the manufacturers’ instructions. The enzymes required for the RT-RPA reaction were supplied as freeze-dried powder contained within tubes, which were added with 29.5 μL of rehydration buffer, 2.1 μL of each primer (10 μm), 1 μL of the template, and water to reach a final volume of 47.5 μL. Then, 2.5 μL of magnesium acetate (280 mM) was added into the tube followed by transient centrifugation. The tubes were placed in a metal bath and incubated at 37 °C for 30 min.

### 2.6. PfAgo Cleavage Assays

A PfAgo reaction was set up in an 80 μL volume, incorporating 20 μL total RT-RPA reaction products, 2 μM of the purified PfAgo, and 2 μM of 5′-phosphorylated gDNA. The reaction mixture was then supplemented with 8 μL of a 10× reaction buffer, composed of 200 mM HEPES (pH 7.5), 2.5 M NaCl, and 0.8 mM MnCl_2_. The reaction mixture was incubated for 30 min at 95 °C, and was followed by an analysis of the resultant product via 3% TAE gel.

### 2.7. PfAgo Detection Assays

A PfAgo reaction mixture with a total volume of 20 μL was prepared by mixing 2 μL of 10× Reaction Buffer, 2 μM of PfAgo, 1 μM ssDNA probe, and 0.8 mM of MnCl_2_, followed by adding this mixture to 5 µL of RT-RPA product. To detect single-target nucleic acid, the tube was incubated at 95 °C for 30 min. The products were then visualized using a blue light transilluminator. For multi-target detection, the reaction tube was subjected to a BioRad CFX384 Real-Time PCR machine(Bio-Rad, Hercules, CA, USA)and incubated at 95 °C, and this was followed by the recording of fluorescence signals every minute over 30 min.

## 3. Result and Discussion

### 3.1. Principle of the RT-RPA-PfAgo Method

**Figure 1:**
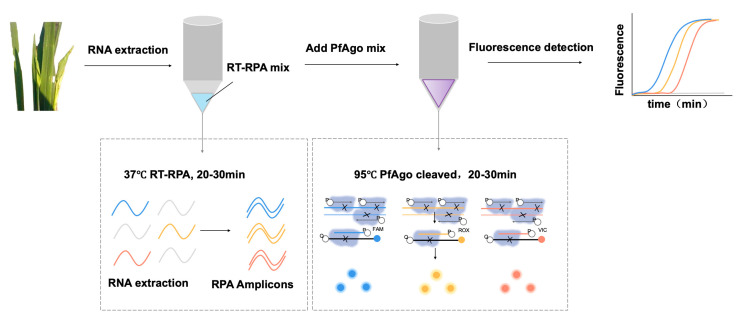
Schematic illustration of RT-RPA-PfAgo for simultaneous detection of three RNA targets.

The mechanism underlying RT-RPA-PfAgo detection, as illustrated in Figure 1, initiates with the RT-RPA of specific fragments: S8gp1 gene fragment (nt 684-987) from RRSV segment 8 (GenBank Accession NC_003758.1), the ORF1 gene fragment (nt 925-1250) from RBSDV segment 10 (GenBank Accession NC_003733.1), and the Pc5 gene fragment (nt 1614-1892) from RGSV segment 5 (GenBank Accession NC_003733.1). The amplified fragments were then specifically identified by PfAgo protein under the guidance of 5′-phosphorylated gDNA. The PfAgo triggered cleaving phosphodiester bonds between the 10th and 11th bases of the target DNA from the 5′ end upon base pairing between the gDNA and one strand of the RPA amplicon, which resulted in the formation of a new 5′-phosphorylated ssDNA, with an ability to function as guiding gDNA for a subsequent PfAgo cleavage, targeting a synthetically designed single-stranded DNA probe labeled with fluorophore and a quencher groups on its termini. The FAM and BHQ1 were held near due to their complementary nature due to the hairpin-like structure of probe terminal sequences. The loop sequence of the probe was engineered to pair with the newly generated 5′-phosphorylated ssDNA, thereby initiating specifically probe cleaving by PfAgo, liberating the fluorophore, which was subsequently detected fluorometrically. The distinct secondary gDNA produced by varying target fragments directs PfAgo to cleave specific probes with different fluorescent labels, enabling concurrent detection of multiple targets within a singular reaction. This dual recognition and cleavage process augments the method’s specificity and accuracy, significantly reducing potential false-positive results.

### 3.2. Establishing the Workflow for RRSV Detection via RT-RPA-PfAgo

**Figure 2:**
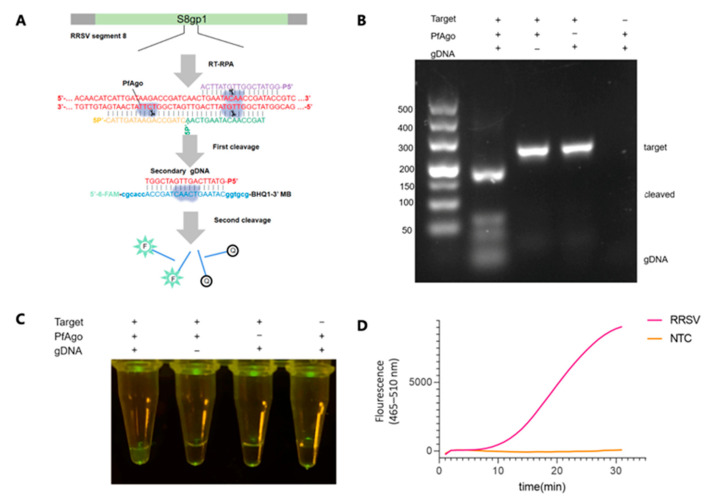
RSV Detection via RT-RPA-PfAgo. (**A**) Schematic representation of gDNAs and the designated probe tailored for RRSV detection. The PfAgo cleavage area and the newly formed secondary gDNA are highlighted in red. Three 5-phosphorylated single-stranded DNA guides are depicted in shades of purple, green, and yellow. Molecular beacons are represented and emphasized in blue. (**B**) Illustration of PfAgo-mediated cleavage activity on the RT-RPA-derived amplicon, as visualized on a 3% TAE gel. (**C**) Visual fluorescence of positive samples under blue light transilluminator. (**D**) Fluorescence detection of RRSV by the RT-RPA-PfAgo method is shown as a fluorescence curve. NTC: non-template control.

The coding region from 684 to 987 nt of the S8gp1 gene (GenBank Accession NC_003758.1) was targeted explicitly within the RRSV genome for amplification via RT-RPA, employing the primer pair (RRSV-RPA-F/RRSV-RPA-R). The results from TAE gel electrophoresis displayed bands of the expected size, confirming the efficacy of our RPA primer design (Figure 2B). Three gDNAs (RRSV-g1, -g2, and -g3) were designed within this region to guide PfAgo in generating RRSV-specific secondary gDNA (RRSV-secondary-g4), as detailed in Figure 2A and Table S1. The cleavage activity of the resultant PfAgo/guide complexes against dsDNA targets confirmed cleavage at the anticipated sites, as shown in Figure 2B. The ability of secondary gDNA to diagnose RRSV nucleic acids was further validated by incorporating PfAgo protein, guide DNAs, and a probe (RRSV-Probe) directly into the RT-RPA amplified products. A visual examination under blue light transmission revealed a distinct green fluorescence, starkly contrasting with the negative control (Figure 2C), which underscores the production of secondary gDNA and the effective guidance of PfAgo in cleaving the RRSV-Probe. Simultaneously, real-time fluorescence quantification demonstrated significant fluorescence signals, as illustrated in Figure 2D and Figure S2. Taken together, these results affirm the effectiveness of the RT-RPA-PfAgo method in detecting RRSV genes and highlight its precision and potential applicability in the field of viral diagnostics.

### 3.3. Optimization of RT-RPA-PfAgo Reaction

**Figure 3:**
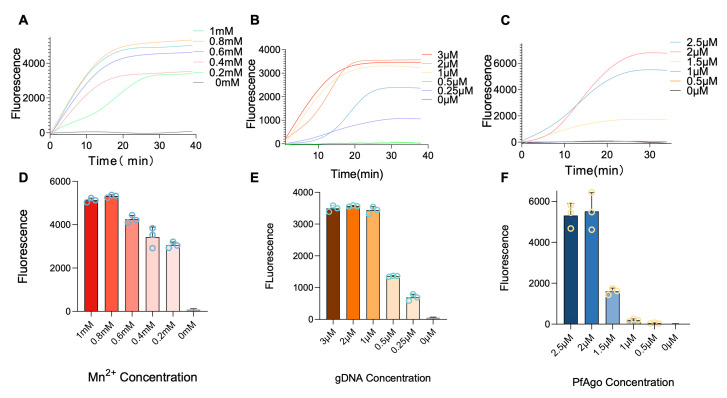
Optimization of the RT-RPA-PfAgo. (**A**–**C**) plots illustrating the temporal accumulation of fluorescence intensity, with each plot corresponding to different concentrations of Mn^2+^ gDNA and PfAgo. (**D**–**F**) Bar charts depicting the endpoint fluorescence signal, with varying concentrations of Mn^2+^ gDNA and PfAgo. Error bars are included to indicate the standard error across three replicates. The RRSV RNA standard, with a concentration of 10^4^ copies per reaction, was utilized as the template in these experiments.

To enhance the RT-RPA-PfAgo reaction’s efficiency, the concentrations of key components, i.e., Mn^2+^, gDNA, and PfAgo, were optimized. The results indicated that Mn^2+^ at a concentration of 0.8 µM yielded the most favorable results among all tested concentrations (1, 0.8, 0.6, 0.4, and 0.2 μM, Figure 3A,D). Similarly, a gDNA concentration of 2 μM concentration of gDNA among all tested concentrations (0.25, 0.5, 1, 2, and 3 μM) was found to be optimal (Figure 3B,E). The PfAgo was evaluated at different concentrations of 0.5, 1, 1.5, 2, and 2.5 μM, where 2 μM of PfAgo was identified to be the most effective concentration (Figure 3C,F). Peak fluorescence values under optimized conditions consistently manifested within 30 min following PfAgo cleavage, resulting in selecting a 30 min duration for all subsequent reactions.

### 3.4. Simultaneous Detection of RRSV, RGSV, and RBSDV Using the RT-RPA-PfAgo System

**Figure 4:**
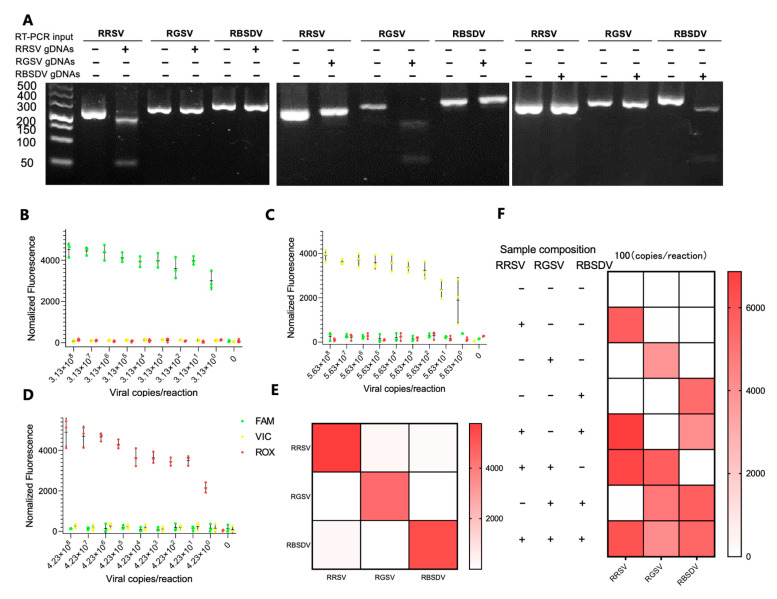
Evaluation of the triplex detection and classification capability of the RT-RPA-PfAgo assay. (**A**) The cleavage mediated by PfAgo on the amplicon derived from RT-RPA, as observed on a 3% TAE gel. (**B**–**D**) The Limit of Detection (LoD) assay for triplex fluorescence analysis using serially diluted RRSV, RGSV, and RBSDV, respectively. The error bars in the figure represent the standard deviation calculated from three replicates. (**E**) A heatmap depicting the orthogonal specificity of RT-RPA-PfAgo fluorescence detections for RRSV, RGSV, and RBSDV. (**F**) A triplex fluorescence analysis was conducted using a mixture of RNA targets. The color intensity of the heatmaps corresponds to the average fluorescence intensity observed in three replicates.

The RT-RPA-PfAgo methodology was further refined and enhanced based on the specific guide-directed cleavage capability of PfAgo, enabling multiplex detection for viral RNA targets. Three specific gene fragments, i.e., S8gp1 from RRSV (nt 684-987), the ORF1 from RGSV (nt 925-1250), and the Pc5 from RBSDV (nt 1614-1892), were targeted. The multiplex viral nucleic acid detection was achieved by designing multiplex RT-RPA primers, primary gDNAs, and corresponding probes (RRSV-FAM, RGSV-VIC, and RBSDV-ROX, see Table S1 and Figure S3). The results of RT-RPA and PfAgo cleavage assays revealed the effectiveness of our RPA primer design. Additionally, the RPA products were cleaved explicitly by their respective gDNAs, with no evidence of non-specific cleavage by non-complementary gDNAs (Figure 4A), which was further corroborated by exclusive fluorescence capture in the presence of the RNA template, probes, and corresponding gDNAs, cementing the method’s precision (Figure 4E). The method sensitivity was evaluated by subjecting standard RNA samples of RRSV, RGSV, and RBSDV to 10-fold gradient dilutions across seven magnitudes. The results were encouraging when the method detected a low concentration of 3.13 copies/μL for RRSV, 4.13 copies/μL for RGSV, and 5.13 copies/μL for RBSDV within 50 min (Figure 4B–D). Noteworthily, the method detected single, double, and triple targets in a single reaction (Figure 4F and Figure S4), with no off-target signals emerging in any reaction combinations, and the signal intensities in the multiplex reactions remained uniform.

### 3.5. Field Sample Analysis via RT-RPA-PfAgo

**Figure 5:**
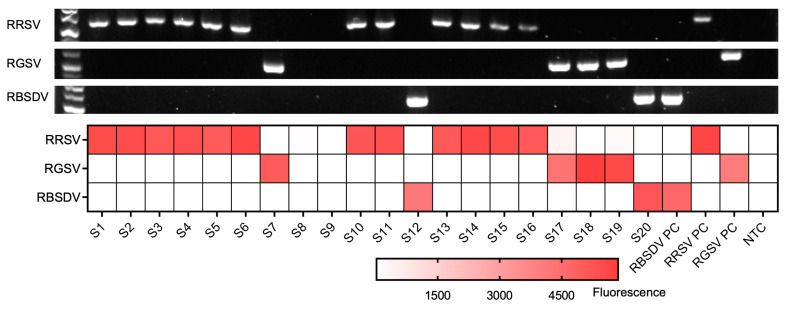
Comparison between the RT-RPA-PfAgo assay and RT-PCR results for field samples. The top three rows display the RT-PCR detection results, while the heatmap illustrates the fluorescence results obtained from the RT-RPA-PfAgo method. S1–S22 represents each field sample. The positive control is denoted as “PC,” and the non-template control is indicated as “NTC”.

To assess the efficacy of the triplex RT-RPA-PfAgo assay on field samples, 22 rice leaf tissue samples, which were previously tested for RRSV, RBSDV, and RGSV infection via RT-PCR (Figure 5), were collected from various regions in Hunan, Zhejiang, and Shanghai, China, between 2019 and 2023,. By employing the RT-RPA-PfAgo method, we successfully identified 17 samples as RRSV-positive, 2 as RGSV-positive, and 1 as RBSDV-positive. Our findings demonstrated a complete concordance (100%) between the RT-RPA-PfAgo and RT methods (Figure 5). These results advocated and highlighted the capability of the multiplex RT-RPA-PfAgo assay to accurately differentiate between various viral species found in field samples, emphasizing its potential applications in the diagnosis of plant viruses.

## 4. Conclusions

In summary, utilizing the RT-RPA-PfAgo system effectively exploits PfAgo’s unique specificity in identifying RRSV, RGSV, and RBSDV. This innovative approach provides a dependable solution for the simultaneous detection of diverse rice viruses, showcasing expeditious detection, higher sensitivity, and accurate differentiation between distinct viral sequences. With a sensitivity range from 3.13 to 5.13 copies/μL and complete agreement with RT-PCR results, the RT-RPA-PfAgo system emerges as a robust, specific, and highly sensitive tool, holding considerable potential in future plant virus diagnostics.

## Data Availability

Not applicable.

## Supplementary Materials

The following supporting information can be downloaded at: https://www.mdpi.com/article/10.3390/bios13100941/s1, Figure S1: Analyzing PfAgo with SDS-PAGE after purification; Figure S2: Endpoint fluorescence signal for RRSV detection; Figure S3: Diagram illustrating the gDNAs and specific probes designed for detecting RGSV and RBSDV. Figure S4: Evaluation of the multiplex detection capability of the RT-RPA-PfAgo assay; Table S1: List of primers, gDNAs and probes used in this study.
