# Supplementary material for: RT-RPA-PfAgo System: A Rapid, Sensitive, and Specific Multiplex Detection Method for Rice-Infecting Viruses
Source: Biosensors (Basel). 2023 Oct 20;13(10):941. doi: 10.3390/bios13100941 (PMC10605773; doi:10.3390/bios13100941)
Supplement: Supplementary file 1 [file biosensors-13-00941-s001.zip › biosensors-2630714-supplementary.pdf]

Table S1 List of primers, gDNAs and probes used in this study.

Target	Prime	Sequence(5'-3')	Usage
**RRSV**	RRSV-t7-F	TaatacgactcactatagggATCCATCGACTTGGTTTAGCCAA[a]	PCR amplification of templates for in vitro transcription
	RRSV-t7-R	ATAGGATTAGTGATGCTTCCACAG	
	RRSV-RPA-F	CGAATCATCACTGAACAAGTATTTGGAGCT	RPA amplification of target sequence
	RRSV- RPA-R	ATTGACGAGTCCTCTGGCGGAATGGATGGT	
	RRSV-g1	P-CATTGATAAGACCGATC[b]	Guide pfAgo to a specific target sequence that subsequently cleaved by pfAgo
	RRSV-g2	P-AACTGAATACAACCGAT[b]	
	RRSV-g3	P-GGTATCGGTTGTATTCA[b]	
	RRSV-secondary-g4	P- GTATTCAGTTGATCGGT[b]	gDNA produced from first round cleavage
	RRSV-Probe	FAM-cgcaccACCGATCAACTGAATACggtgcg-BHQ1[c]	Molecular beacon
**RGSV**	RGSV-t7-F	taatacgactcactatagggAGCATCCTTCAGATCAGCTGTAT[a]	PCR amplification of templates for in vitro transcription
	RGSV-t7-R	CTAAAGCTCAGAAGGATAAGCACA	
	RGSV- RPA-F	TTTTATGCACTCAATAGCTAATCTGAACAG	RPA amplification of target sequence
	RGSV- RPA-R	ACACTAAGTCAGCAATGATAACACACTGCA	
	RGSV-g1	P-GAGATCATCCTTCTACC[b]	Guide pfAgo to a specific target sequence that subsequently cleaved by pfAgo
	RGSV-g2	P-AGCTATCTTATCATCA[b]	
	RGSV-g3	P-TGTCTGATGATAAGATA[b]	
	RGSV-secondary-g4	P-TAAGATAGCTGGTAGAA [b]	gDNA produced from first round cleavage
	RGSV-Probe	VIC-cgcaccTTCTACCAGCTATCTTA ggtgcg- BHQ1 [c]	Molecular beacon
**RBSDV**	RBSDV-t7-F	taatacgactcactatagggCATGGCAGGTTAAATCTAAAGTT[a]	PCR amplification of templates for in vitro transcription
	RBSDV-t7-R	CGCTCAACACTTCGCCAATTTTAC	
	RBSDV- RPA-F	TGATGATCCAGATGAATATGAATTGACCA	RPA amplification of target sequence
	RBSDV- RPA-R	CATTTCGCCTTTATGAGTTTGAACTACAAC	
	RBSDV-g1	P-CACGTTGCGCACTAATT[b]	Guide pfAgo to a specific target sequence that subsequently cleaved by pfAgo
	RBSDV-g2	P-ACGATGGCACCTCTGCT[b]	
	RBSDV-g3	P-AGAAGCAGAGGTGCCAT[b]	
	RBSDV-secondary-g4	P-GTGCCATCGTAATTAGT[b]	gDNA produced from first round cleavage
	RBSDV-Probe	ROX-cgcaccACTAATTACGATGGCAC ggtgcg- BHQ2 [c]	Molecular beacon

[a] The sequence shown in lower case letters represent the T7 promoter sequence
[b] 5'-phosphorylated ssDNA
[c] Lowercase letters represent hairpin sequences in probe

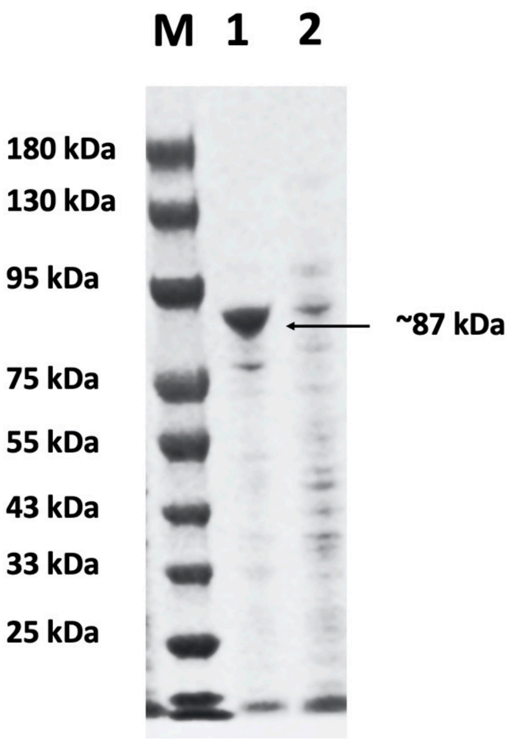

Figure S1: Analyzing PfAgo with SDS-PAGE after purification. M: pre-stained protein marker Lane1: purified PfAgo; Lane2: flow-through; The PfAgo protein band was indicated with a black arrow.

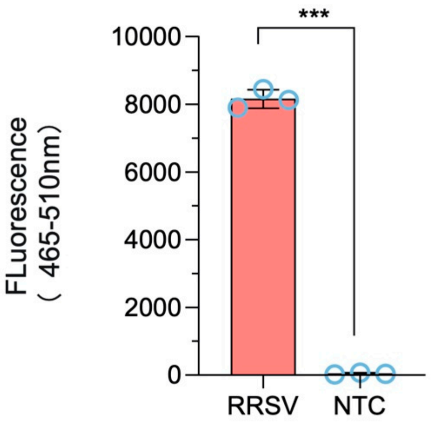

Figure S2: Endpoint fluorescence signal for RRSV detection. NTC: non-template control. ***P < 0.001.

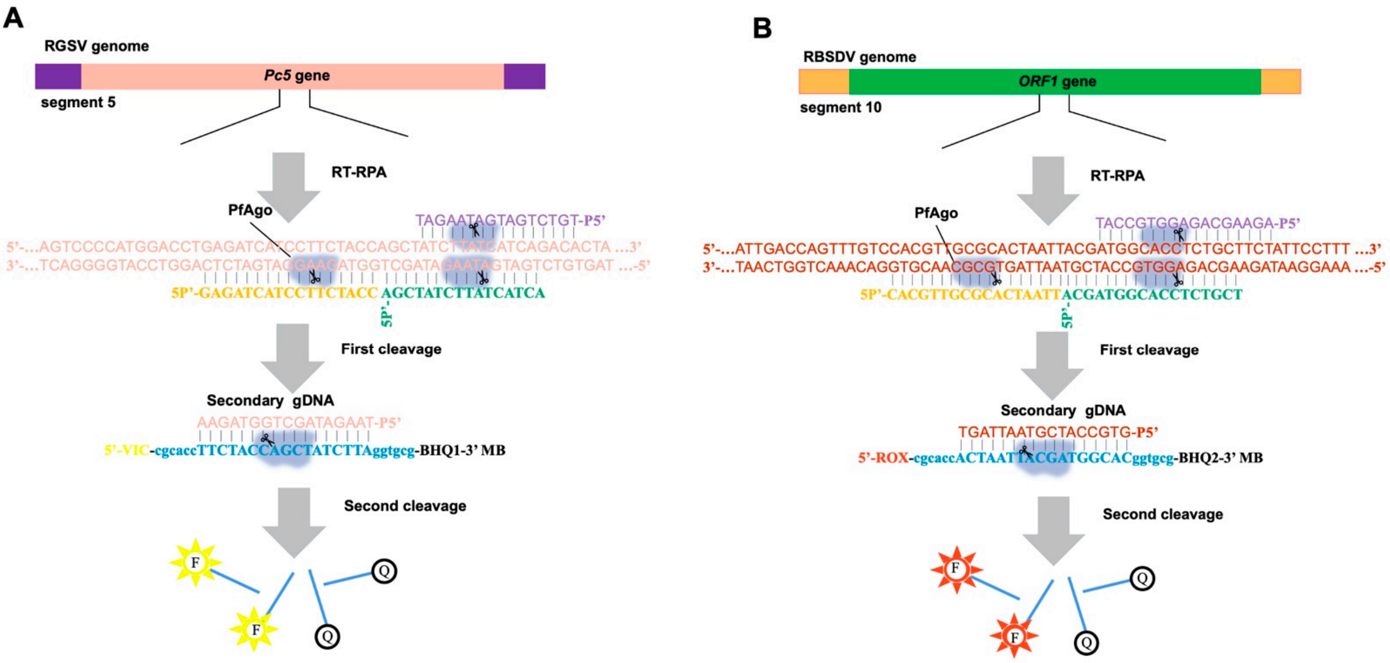

Figure S3: Diagram illustrating the gDNAs and specific probes designed for detecting RGSV (A) and RBSDV (B). Three 5-phosphorylated single-stranded DNA guides are depicted in shades of purple, green, and yellow. Molecular beacons are represented and emphasized in blue.

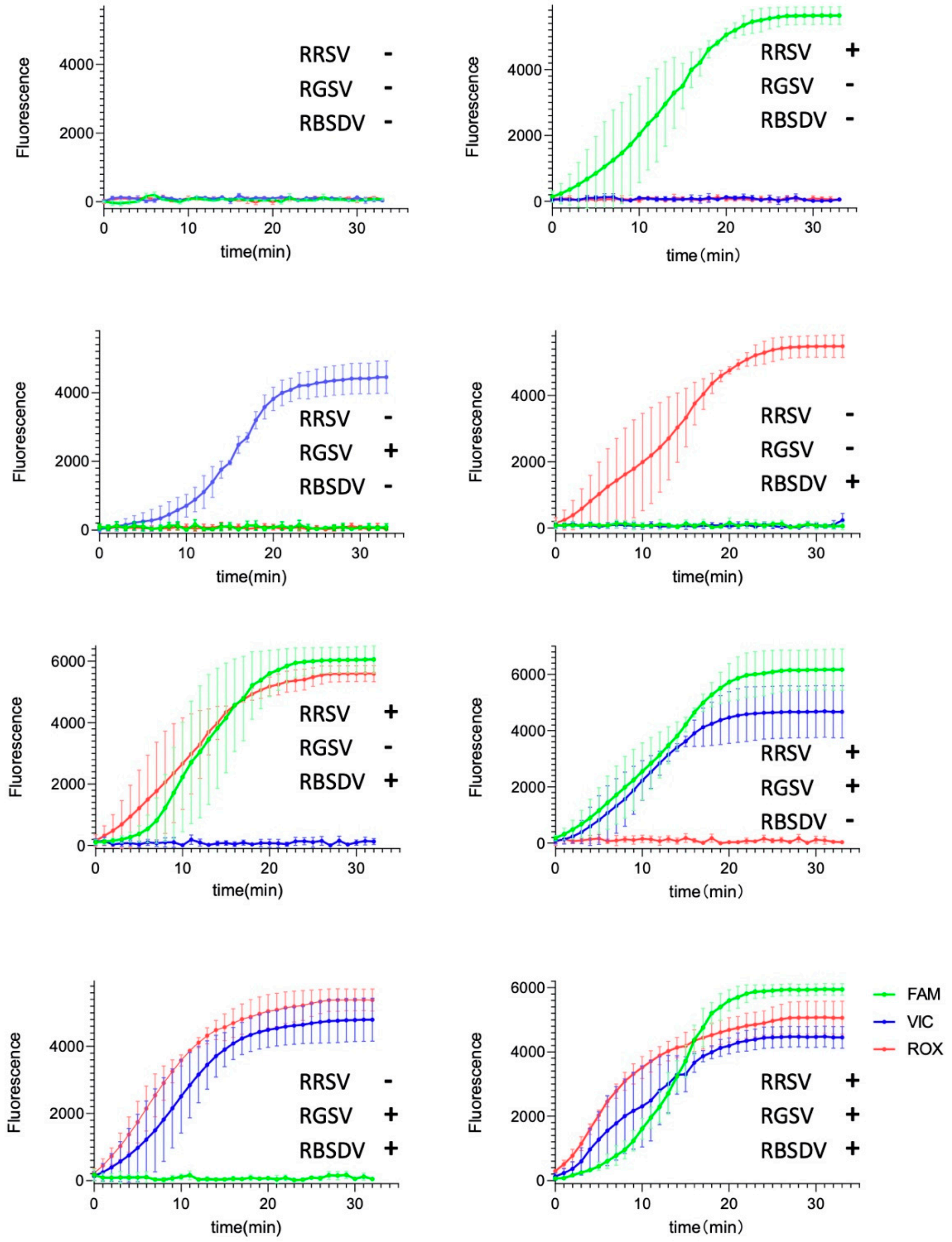

Figure S4: Evaluation of the multiplex detection capability of the RT-RPA-PfAgo assay. The assay's capacity to detect RRSV, RGSV, and RBSDV simultaneously was evaluated by utilizing single, double, and triple mixtures comprising 100 copies of reference RNA from each virus. The fluorescence curve obtained by applying the RT-RPA-PfAgo method is evidence for successfully identifying RNA targets.
